# Parliament and the Profession

**Published:** 1917-12-15

**Authors:** 


### Parliament and the Profession.

### Medical Boards under National Service.

SIR A. GEDDES stated that members of medical boards are appointed by the Chief Commissioner of Medical Services on the authority of the Minister of National Service. The boards are composed of civilian medical practitioners drawn from a panel set up in the area of each board. The chairman of each board, who is the Deputy-Commissioner of Medical Services of the Ministry for an area, arranges with the local medical war committee a rota of attendances of members at sessions of his board. The panels are formed from medical practitioners whose names have been suggested by the local medical war committee; a board of four requires a panel of eight to twelve members; the nominations reach the Ministry through the Central Medical War Committee, they are reviewed in the Medical Department of the Ministry and by the Chief Commissioner of Medical Services for the region to which the panel belongs, and finally the appointments to the panel are made by the Minister and notified to the Deputy-Commissioner of the area.

The Central Medical War Committee is the statutory appeal tribunal for the medical profession, and any member of a National Service medical board called upon to undertake military service may have his case reviewed by this appeal tribunal.

### Inoculation in the Navy.

THE Secretary to the Admiralty (Dr. Macnamara) informed Mr. Dundas White that inoculation in the Royal Navy is not compulsory. Certain consequences, however, ensue from refusal on the part of any individual to be inoculated against infectious disease. In particular, such individuals would be debarred from landing in ports where there may be any danger of contracting any disease against which inoculation is regarded by the Admiralty as advisable.

### Medical Volunteer Corps.

MR. MACPHERSON informed Sir William Collins that sixteen County Medical Volunteer Corps have been formed, or are in process of formation. The total number of field ambulances provided by those corps is twenty-nine. No Medical Volunteer Corps has been formed for the County of London, but it may be possible to utilise the services of certain *personnel* in the County of London if developments, which are under consideration, mature.

### Wounded Soldiers and Restaurants.

IN reply to a question put by Mr. Jowett, in which it was suggested that hardship arises through a wounded soldier not being allowed to enter a licensed restaurant, Mr. Macpherson pointed out that it is a concession to soldiers to be allowed out of hospital which would not be extended to civil patients. Intoxicants are only allowed to patients in hospital on medical orders, and it is evident that they cannot be allowed to obtain them outside hospitals; moreover, there are plenty of unlicensed restaurants where meals can be served.

### A Hospital Workers' Union.

MR. C. DUNCAN asked the Under-Secretary of State for War whether he is aware that excitement and unrest exist amongst the women cleaners employed at the King George Hospital owing to the dismissal of the secretary of the branch of the union to which the women belong. Mr. Forster said that he knew of no unrest, and that the woman in question was amongst a number discharged when a reduction in the staff was found possible, her position not being ascertained until after the event.

### Supply of Nurses Committee and V.A.D.s.

MR. MACPHERSON stated that the bulk of the recommendations of the Supply of Nurses Committee had been carried out, and that he hoped soon to supply members of Parliament with a statement as to outstanding points.

### Pharmaceutical Preparations.

IN reply to a question put to the President of the Local Government Board as to the action of the British Medical Association and the Pharmaceutical Society in issuing a formulary containing substitutes for glycerine and sugar, it was stated that the attention of public analysts had been drawn to the fact, and that the President had been advised that the omission of sugar and glycerine from the preparations in question does not materially lessen their value as medicines.

A question on the same point, addressed to the National Health Insurance Commissioners, in view of the interests of insured persons, elicited the answer that the alternative preparations do not require the approval of the Insurance Commissioners. It was pointed out that it is the duty of every doctor attending an insured patient to prescribe the proper and sufficient drugs and medicines necessary for the efficient treatment of the case, and this is in no way affected by the issue of the formulary in question.

### Dispensers in Military Hospitals.

MR. MACPHERSON informed Mr. Hohler that all dispensers in military hospitals are required to be qualified under the conditions prescribed in the Standing Orders for the Royal Army Medical Corps. Female dispensers are employed wherever possible, and there is no information of any unqualified male dispensers being employed.

